# Interferon α Induces the Apoptosis of Cervical Cancer HeLa Cells by Activating both the Intrinsic Mitochondrial Pathway and Endoplasmic Reticulum Stress-Induced Pathway

**DOI:** 10.3390/ijms17111832

**Published:** 2016-11-02

**Authors:** Wei-Ye Shi, Cheng Cao, Li Liu

**Affiliations:** 1 Department of Microbiology, Institute of Basic Medical Sciences, Chinese Academy of Medical Sciences & Peking Union Medical College, Beijing 100005, China; feiwudeyezi@126.com; 2 Beijing Institute of Biotechnology, Academy of Military Medical Sciences, 27 Taiping Rd., Haidian District, Beijing 100850, China; cao_c@sohu.com

**Keywords:** interferon α (IFN-α), apoptosis, HeLa cell, intrinsic mitochondrial pathway, ER stress, caspase 4

## Abstract

The interferon α (IFN-α) has been often used as a sensitizing agent for the treatment of various malignancies such as hepatocellular carcinoma, malignant melanoma, and renal cell cancer by promoting the apoptosis of thesetumor cell types. However, the effect of IFN-α on cervical cancer remains unknown. In this study, HeLa cells were used as a testing model for the treatment of IFN-α on cervical cancer. The results indicate that IFN-α markedly inhibits the proliferation and induces the apoptosis of HeLa cells. The activation of caspase 3, the up-regulation of both Bim and cleaved poly (ADP-ribose) polymerase (PARP) 1, the down-regulation of Bcl-xL, as well as the release of cytochrome c from mitochondria were significantly induced upon IFN-α treatment, indicating that the intrinsic apoptotic pathway could be activated by IFN-α treatment. In addition, caspase 4—which is involved in the endoplasmic reticulum (ER) stress-induced apoptosis—was activated in response to IFN-α treatment. Knocking down caspase 4 by small interfering RNA (siRNA) markedly reduced the IFN-α-mediated cell apoptosis. However, no significant changes in the expressions of caspases 8 and 10 were observed upon IFN-α treatment, indicating that the apoptosis caused by IFN-α might be independent of the extrinsic apoptotic pathway. These findings suggest that IFN-α may possess anti-cervical cancer capacity by activating cell apoptosis via the intrinsic mitochondrial pathway and caspase-4-related ER stress-induced pathway.

## 1. Introduction

Human interferon α (IFN-α) belongs to the type I interferon (IFN-I) that are widely known to induce potent innate immune response against both viral infection and cancers [1,2,3]. IFN-α exerts its anticancer effects by inhibiting cell proliferation, promoting cell apoptosis, and/or suppressing oncogene expression [4,5,6,7,8,9]. IFN-α has been increasingly used in clinics to treat a wide range of malignancies such as hepatocellular carcinoma (HCC), malignant melanoma, and renal cell cancer (RCC) [10,11,12,13,14]. However, IFN-α has been barely used in the treatment of cervical cancer either alone or in combination with other agents. Although cervical cancer is the leading threat to women’s health worldwide, there is no cure to this disease at present [15,16]. Therefore, finding a novel and effective treatment for this disease is highly demanded and should be the direction of scientist’s efforts.

Apoptosis is the basic physiological process of the body to eliminate either the damaged, aged, or mutated cells to maintain homeostasis of the body system [17,18,19,20]. Cell apoptotic signals can be generated from and transmitted through different cellular compartments such as mitochondria, endoplasmic reticulum (ER), or the death receptors on cell surfaces [21,22]. Although induced by different means, apoptosis is primarily executed by a family of proteases known as the caspases (cysteinyl, aspartate-specific proteases), and among them the activation of caspase 3 is the hallmark of apoptosis. The apoptotic signal may target the outer membrane of mitochondria to increase membrane permeability, which in turn promotes the release of cytochrome c [22]. The apoptotic signal may be generated from the ER by various stresses in a caspase 4 dependent and independent manner. In addition, the membrane proximity-induced activations of caspases 8 and 10 release the respective cleaved caspase molecules within the cytoplasm [23,24,25,26]. Subsequently, the three pathways stimulate the common effectors (caspases 3, 6, and 7) to ultimately execute cell apoptosis.

In this study, HeLa cells were used as a testing model for the treatment of IFN-α on cervical cancer. The influence of IFN-α on HeLa cell proliferation and apoptosis was systematically evaluated. We demonstrate that IFN-α-induced cell apoptosis in HeLa cells is mainly due to the activations of both the intrinsic apoptotic pathway and caspase 4/ER stress-induced apoptotic pathway, but independent of the activation of the extrinsic apoptotic pathway.

## 2. Results

### 2.1. Interferon α (IFN-α) Inhibits the Proliferation of HeLa Cells

**Figure 1:**
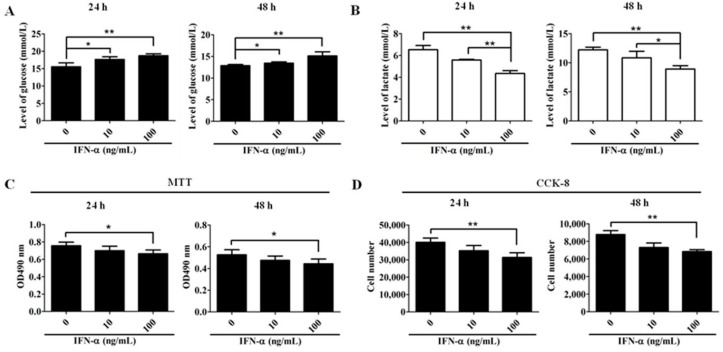
Interferon α (IFN-α) inhibits the proliferation of human cervical cancer cell line, HeLa cells. The detection of glucose (**A**) and lactate (**B**) levels in IFN-α-treated HeLa cell culture medium. HeLa cells were treated with increased doses (0, 10, and 100 ng/mL) of IFN-α for 24 and 48 h, respectively. The glucose and lactate levels presented in cell culture medium were detected by Biosen C-Line. Each value is represented as mean ± SD from three independent experiments; (**C**) MTT (3-(4,5-dimethylthiazol-2-yl)-2,5-diphenyl tetrazolium bromide) analysis on cell proliferation. HeLa cells were treated with increased doses (0, 10, and 100 ng/mL) of IFN-α for 24 and 48 h. The treated HeLa cells were collected and assayed by MTT. The reaction products were measured at 490nm with a plate reader. Each value is represented as mean ± SD from three independent experiments; (**D**) Cell-Counting Kit (CCK)-8 analysis on cell proliferation. After treatment with increased doses (0, 10, and 100 ng/mL) of IFN-α, the treated HeLa cells were collected and assayed by CCK-8 kit. The reaction products were measured at 450 nm with a plate reader. The variable cell number for each dose was calculated against the standard curve. Each value is represented as mean ± SD from three independent experiments. After statistical analysis, results were considered to be significant if *p* < 0.05 (*) or *p* < 0.01 (**).

It has been shown that IFN-α is able to inhibit cell proliferation and to induce cell apoptosis in some cancer cell types [27]. We hypothesized that IFN-α may also possess anti-proliferative and pro-apoptotic effects on HeLa cells. To test this hypothesis, the levels of glucose and lactate of HeLa cell culture medium after IFN-α treatment were determined. Figure 1A,B show that the glucose level increased and lactate level decreased when the delivered doses of IFN-α were enhanced, indicating that the proliferation potential of HeLa cells was inhibited. Consistently, IFN-α treatment on HeLa cells resulted in a marked and dose-dependent reduction on the amount of viable cells, as demonstrated by both MTT (3-(4,5-dimethylthiazol-2-yl)-2,5-diphenyl tetrazolium bromide, Figure 1C) and Cell-Counting Kit (CCK)-8 assays (Figure 1D).

### 2.2. IFN-α Induces the Apoptosis of HeLa Cells

**Figure 2:**
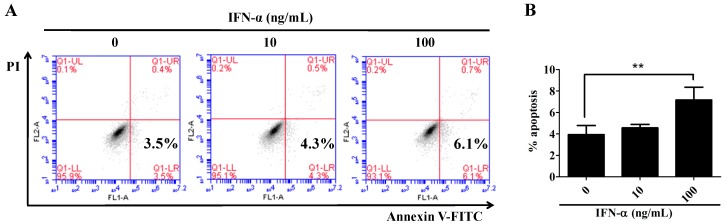

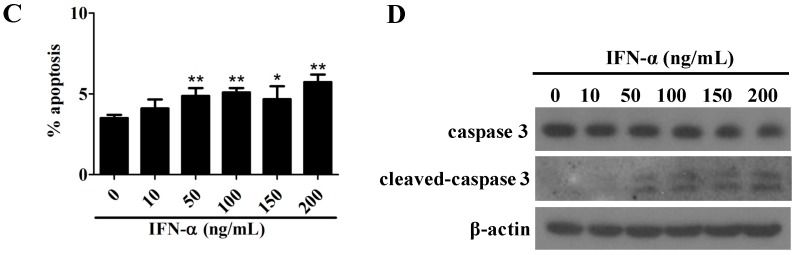
IFN-α promotes apoptosis of HeLa cells. (**A**) Flow cytometric analysis on HeLa cell apoptosis after IFN-α treatment. HeLa cells were first seeded onto a 12-well culture plate and treated with different doses of IFN-α. After 48 h culture, the cells were harvested and subjected to annexin V/propidium iodide (PI) double staining followed by flow cytometric analysis; (**B**) Quantitation of the apoptosis of the HeLa cells after IFN-α treatment. The IFN-α-treated HeLa cells were prepared as described in (**A**) and subjected to annexin V/PI double staining. Each value is represented as mean ± SD from three independent experiments. After statistical analysis, results were considered to be significant if *p* < 0.05 (*) or *p* < 0.01 (**); (**C**) The dose effect of IFN-α-induced apoptosis in HeLa cells. HeLa cells were treated with six increasing doses of IFN-α (0, 10, 50, 100, 150, and 200 ng/mL) for 48 h, then, cell apoptosis was detected with annexin V/PI double staining followed by flow cytometric analysis. Each value is represented as mean ± SD from three independent experiments. After statistical analysis, results were considered to be significant if *p* < 0.05 (*) or *p* < 0.01 (**); (**D**) Western blot analysis on the activation of caspase 3. The whole-cell lysates prepared from (**C**) were probed with anti-caspase 3 and anti-cleaved-caspase 3 antibodies. *β-actin* gene expression is served as an internal control.

Inhibition of cell proliferation often leads to cell apoptosis. To test this possibility, HeLa cells were treated with IFN-α for 48 h and subjected to annexin V and propidium iodide (PI) double staining. Subsequent flow cytometric analysis showed a marked increase in the population of Annexin V+/PI− cells in response to a higher dose of IFN-α delivery (Figure 2A), indicating that early apoptosis is induced upon IFN-α treatment. Statistical analysis showed that a higher dose delivery of IFN-α significantly increased the population of apoptotic cells (Figure 2B). To further define the optimal dose of IFN-α, HeLa cells were treated with six increasing doses (0, 10, 50, 100, 150, and 200 ng/mL) of IFN-α for 48 h. Figure 2C shows that dose-dependent apoptosis was induced as the IFN-α concentration increased from 0 to 50 ng/mL. The result also indicates that IFN-α-mediated cell apoptosis reaches a platform when the delivered dose of IFN-α is more than 50 ng/mL. To further confirm this result, whole-cell lysates were prepared from these IFN-α-treated cells (depicted in Figure 2C) and probed with anti-caspase 3 and anti-cleaved-caspase 3 antibodies, then subjected to Western blot analysis. Figure 2D demonstrates that the increased delivery of IFN-α significantly reduced the pre-caspase3 expressions and markedly increased the cleaved caspase 3 expressions, indicating that IFN-α-induced cell apoptosis indeed reaches a platform after 50 ng/mL. Based on the above information, we chose the concentration 100 ng/mL as the optimal dose for IFN-α treatment in the experiments afterwards.

### 2.3. The Mitochondrial Apoptotic Pathway Is Activated by IFN-α Treatment

**Figure 3:**
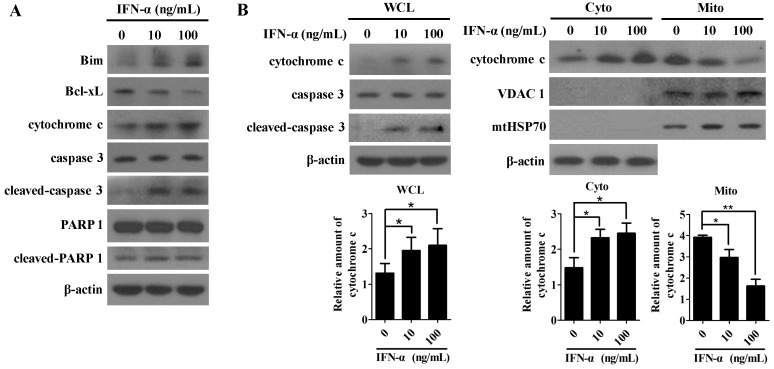
IFN-α-mediated cell apoptosis in HeLa cells is associated with the mitochondrial-mediated intrinsic pathway. (**A**) Western blot analysis of the expressions of the key mediators associated with the intrinsic apoptotic pathway. HeLa cells were treated with IFN-α for 48 h. The whole-cell lysates (WCL) were prepared and probed with anti-Bim, anti-Bcl-xL, anti-caspase 3, anti-cleaved-caspase 3, anti-cytochrome c, anti-PARP1 (poly (ADP-ribose) polymerase 1), and anti-cleaved-PARP1 antibodies. The reaction products were subjected to Western blot analysis. The *β-actin* gene expression is served as an internal control; (**B**) IFN-α promotes cytochrome c release from mitochondria into cytosol in HeLa cells. Mitochondrial (Mito) and cytoplasmic (Cyto) proteins were separated using a mitochondria isolation kit. The whole-cell lysates (WCL) were probed with anti-cytochrome c, anti-caspase 3, and anti-cleaved-caspase 3 antibodies, while the mitochondrial and cytoplasmic lysates were probed with anti-cytochrome c, anti-VDAC1 (voltage-dependent anion channel 1), and anti-mtHSP70 (mitochondrial heat shock protein 70) antibodies. The *β-actin* gene expression is served as an internal control for cytosol. VDAC1 served as a mitochondria loading control. mtHSP70, a mitochondria matrix-specific protein, was included to monitor the quality of the mitochondrial isolation. The diagram at the lower panel represents the relative amounts of cytochrome c in different cell compartments (WCL, Mito, or Cyto) after IFN-α treatment, which were quantitated based on the expressions of either β-actin or VDAC1 from three independent assays using the Image J program. After statistical analysis, results were considered to be significant if *p* < 0.05 (*) or *p* < 0.01 (**).

Next, we asked whether or not IFN-α-induced cell apoptosis is associated with the activation of the intrinsic mitochondria pathway. To address this question directly, cell lysates prepared from IFN-α-treated HeLa cells were probed with anti-Bim, anti-Bcl-xL, anti-caspase 3, anti-cytochrome c, anti-PARP1 (poly (ADP-ribose) polymerase 1), and anti-cleaved-PARP1 antibodies. Bcl-xL belongs to the BCL2 family and exerts its anti-apoptotic function by preventing the oligomerization of Bax/Bak proteins at the mitochondrial outer membrane. However, the other member of BCL2 family, Bim, has the opposite effect by promoting cell apoptosis [28]. Oligomeric Bax/Bak proteins that are inserted into the outer mitochondrial membrane can cause alterations of membrane permeability and the release of cytochrome c, which eventually activates caspase 3 for executing cell apoptosis [29]. Indeed, IFN-α treatment was observed to markedly enhance the expression level of Bim protein, dramatically inhibit the protein expression of Bcl-xL, and significantly activate caspase 3 expression in a dose-dependent manner (Figure 3A). In addition, a slight increase in the production of the cleaved PARP1 was also detected as the doses of IFN-α increased (Figure 3A).

Figure 3B shows that the expression levels of cytochrome c in whole-cell lysates were markedly increased, comparable to the dose-dependent activation of caspase 3, indicating that cytochrome c might be released into cytoplasm from mitochondrion upon IFN-α treatment. To further confirm this result, both the mitochondrial and cytoplasmic fractions of the IFN-α treated HeLa cells were separated using a mitochondria isolation kit. Figure 3B further demonstrates that the expression level of cytochrome c in mitochondria was significantly decreased as the delivered dose of IFN-α increased, while that in the cytoplasm was the opposite. Thus, the data strongly indicates that IFN-α-mediated apoptosis is associated with the activation of the intrinsic mitochondrial pathway in HeLa cells.

### 2.4. IFN-α Activates the ER Stress-Induced Apoptotic Pathway but Not the Extrinsic Apoptotic Pathway in HeLa Cells

**Figure 4:**
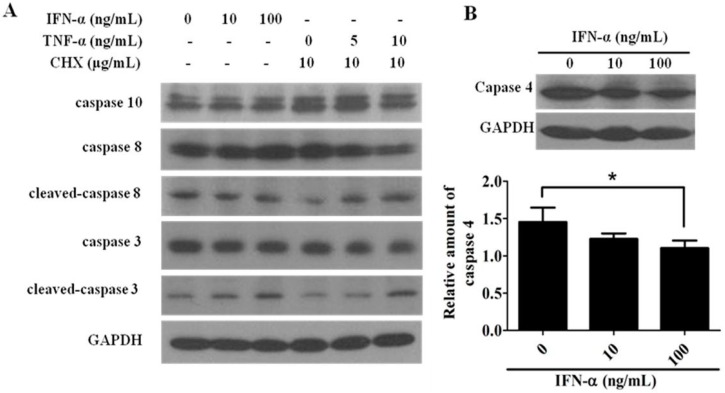
IFN-α mediated cell apoptosis in HeLa cells is also associated with ER stress-induced apoptosis. (**A**) IFN-α does not activate the extrinsic apoptotic pathway. The whole-cell lysates prepared from IFN-α or tumor necrosis factor (TNF)-α/cycloheximide(CHX)-treated HeLa cells were probed with anti-caspase 10, anti-caspase 8, anti-cleaved-caspase 8, anti-caspase 3, and anti-cleaved-caspase 3 antibodies, and subjected to Western blot analysis. *GAPDH* (glyceraldehyde-3-phosphate dehydrogenase) gene expression is served as an internal control; (**B**) IFN-α activates caspase 4-related endoplasmic reticulum (ER) stress-induced apoptosis in HeLa cells. The whole-cell lysates prepared from IFN-α-treated HeLa cells were probed with anti-caspase 4 antibodies and analyzed by Western blot. *GAPDH* gene expression is served as an internal control. The diagram represents the quantitation of the relative caspase 4 protein expression under IFN-α treatment in HeLa cells from three independent assays using Image J program. After statistical analysis, results were considered to be significant if *p* < 0.05 (*).

In addition to the intrinsic apoptotic pathway, the activations of both the extrinsic and ER stress-induced apoptotic pathways might also contribute to IFN-α-mediated cell apoptosis. To test these possibilities, the expressions of caspase 8, 10, as well as caspase 4 were evaluated. As described earlier, the activation of caspases 8 and 10 can turn on the extrinsic apoptotic pathway, while human caspase 4 (equal to mouse caspase 12)—localized on the cytoplasmic side of the ER outer membrane—plays a major role in ER stress-mediated cell death [30,31]. Figure 4A shows that the increased doses of IFN-α did not significantly alter the expression levels of caspases 8/10, while in contrast, caspases 8/10 could be activated by the combined effect of tumor necrosis factor (TNF)-α plus cycloheximide (CHX) in a TNF-α dose-dependent manner. In addition, the activation of caspase 4 could be induced by increased doses of IFN-α (Figure 4B), indicating that the caspase 4-related ER stress-induced apoptotic pathway, but not the extrinsic apoptotic pathway, may also contribute to IFN-α-mediated cell apoptosis in HeLa cells.

**Figure 5:**
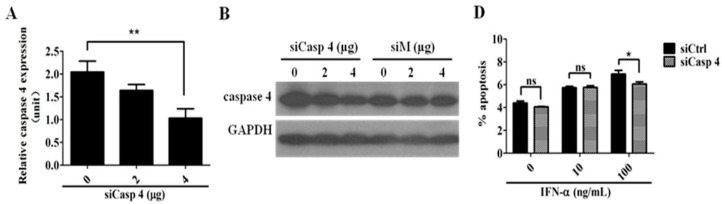

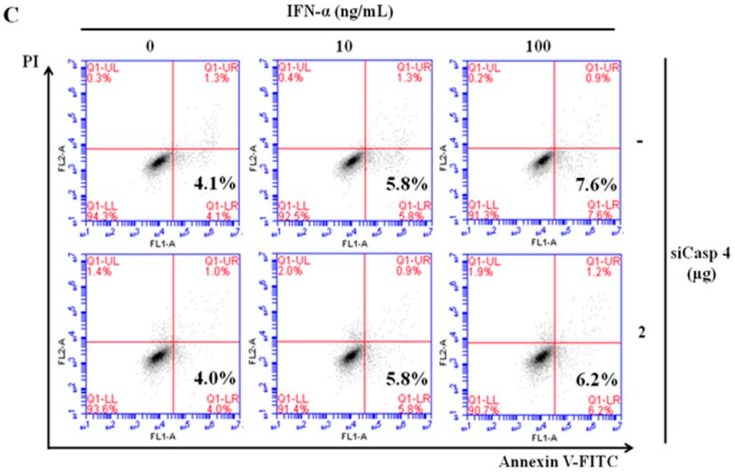
Knocking down the endogenous caspase 4 expression by caspase 4 small interfering RNA (siCasp 4) reduces IFN-α-mediated HeLa cell apoptosis. (**A**) The inhibitory effect of siCasp 4 on endogenous caspase 4 mRNA expression. HeLa cells were transiently transfected with increasing doses (0, 2, and 4 μg) of siCasp 4 for 48 h. Real-time qRT-PCR was conducted to measure the endogenous levels of caspase 4 mRNAs. Each value is represented as mean± SD from three independent experiments. After statistical analysis, results were considered to be significant if *p* < 0.01 (**); (**B**) The inhibitory effect of siCasp 4 on endogenous caspase 4 protein expression. HeLa cells were transiently transfected with increasing doses (0, 2, and 4 μg) of either siCasp 4 or siM (small interfering RNA (siRNA) for SARS-CoV (severe acute respiratory syndrome coronavirus) membrane gene. After 48 h post-transfection, whole-cell lysates were prepared. The reaction products were probed with anti-caspase 4 antibody and subjected to Western blot analysis. *GAPDH* gene expression is served as an internal control; (**C**) HeLa cells were treated with increasing doses of IFN-α in the presence or absence of siCasp4 for 48 h. Cell apoptotic analysis was conducted by annexin V/propidium iodide (PI) double staining followed by flow cytometric analysis; (**D**) Quantitation of apoptotic cells after IFN-α treatment in the presence or absence of 2 μg siCasp 4 as described in (**C**). Each value is represented as mean ± SD from three independent experiments. After statistical analysis, results were considered to be significant if *p* < 0.05 (*) or not significant (ns) if *p* > 0.05.

To further verify the result, caspase 4 small interfering RNA (siCasp 4) was constructed for knocking down the endogenous caspase 4 expressions in HeLa cells. Figure 5A,B demonstrate the specific inhibition of siCasp4 on the targeted gene expressions at both mRNA and protein levels, respectively. To test whether or not the caspase 4-related ER-stress pathway contributes to IFN-α-mediated apoptosis, HeLa cells were transiently transfected with either control or siCasp 4 along with increasing doses (0, 10, 100 ng/mL) of IFN-α. Cell apoptotic analysis shows that a significant rescue of IFN-α-mediated HeLa cell apoptosis by siCasp 4 can be observed when a higher dose (100 ng/mL) of IFN-α was used (Figure 5C,D). All these data articulate that the ER stress-induced apoptotic pathway is also activated by IFN-α treatment in HeLa cells.

### 2.5. The ER Stress-Induced Apoptotic Pathway and the Mitochondrial Apoptotic Pathway Are Independently Induced by IFN-α

**Figure 6:**
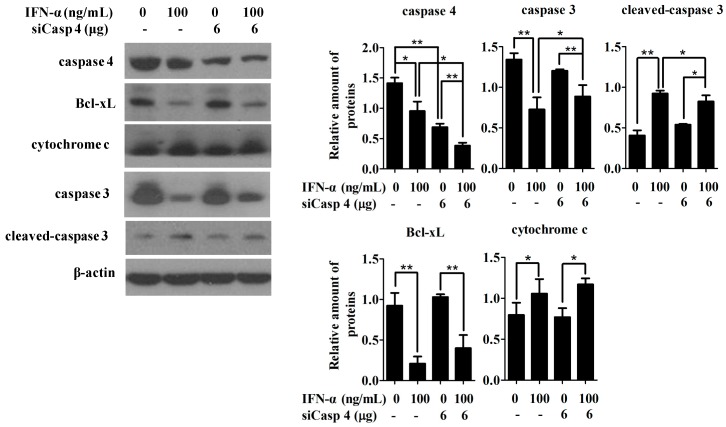
Inhibiting endogenous caspase 4 by siCasp 4 weakens the caspase 3 cleavage, but does not block IFN-α-mediated activation of the mitochondrial apoptotic pathway. HeLa cells were transiently transfected with either control siRNA or siCasp 4 in the presence or absence of 100 ng/mL IFN-α. After 48 h incubation, the whole-cell lysates were harvested, probed with anti-caspase 4, anti-Bcl-xL, anti-cytochrome c, anti-caspase 3, and anti-cleaved-caspase 3 antibodies, and subjected to Western blot analysis (left panel). *β-actin* gene expression is served as an internal control. Quantitation of the relative gene expression of the individual band detected by Western blot was performed using the Image J program. Each bar is represented as mean ± SD from three independent experiments that have been adjusted based on β-actin expressions. After statistical analysis, results were considered to be significant as *p* < 0.05 (*) or *p* < 0.01 (**).

To investigate the interrelationship between the ER stress-induced apoptotic pathway and mitochondrial apoptotic pathway during IFN-α-mediated apoptosis, HeLa cells were transiently transfected with either control siRNA or siCasp 4 in the presence or absence of IFN-α treatment (100 ng/mL). The whole-cell lysates were collected at 48 h post-transfection and subjected to Western blot analysis. Despite the fact that knocking down caspase 4 markedly reduced IFN-α-mediated cell apoptosis (Figure 5C,D), inhibition of caspase 4 expression barely affected the IFN-α-mediated mitochondrial apoptotic pathway, as indicated by the fact that the addition of siCasp 4 did not rescue IFN-α-mediated Bcl-xL repression, nor did it inhibit the up-regulation of cytochrome c; however, it markedly reduced the cleavage of caspase 3 (Figure 6). Collectively, these data indicate that both the caspase 4-related ER stress-induced apoptotic pathway and mitochondrial intrinsic apoptotic pathway are independently activated by IFN-α in HeLa cells.

## 3. Discussion

Cervical cancer is considered to be the second most common malignancy among women worldwide. Currently, there is no cure to this disease, especially to high-risk patients infected by high-risk human be considered ideal, simply due to the adverse reproductive consequences and the presence papillomaviruses (HPVs) such as HPV16 and HPV18. Standard treatments such as ablation and cervical conization are the common strategies for early cervical cancer therapy, but are not recommended for high-risk HPV-infected patients [32]. In addition, most of the cervical cancer patients also receive radiotherapy and chemotherapy either solely or in combination. However, none of these approaches should of persistent HPV infection in the remaining cancer tissues after classic therapy [33]. More recently, the immunotherapy approach, with the help of immunomodulators, has attracted more attention, since this approach can enhance HPV-specific cellular immune responses that might be critical for eliminating HPV-specific neoplasia [16,34]. In this study, we demonstrate that IFN-α alone may possess anticancer capacity by inducing the apoptosis of cervical cancer cells via the intrinsic and ER-stress related apoptotic pathways.

IFN-α has been used as a sensitizing agent for the treatment of numerous malignant human cancers such as renal cell cancer [35], gastric cancer cells [36], malignant melanoma [37], as well as hepatocellular carcinoma (HCC) [9,38], and is most often associated with the stimulation of the extrinsic apoptotic pathway [13,37]. Although the observation that IFN-α can promote cell apoptosis in cervical cancer cells has been documented before [39,40], the detailed mechanism controlling IFN-α-induced cell apoptosis is less clear. Here, we first systematically address this issue by using IFN-α-treated HeLa cells as a study model.

Apoptotic signaling can be generated from cell membrane, mitochondria, and ER. We showed that IFN-α treatment on HeLa cells not only results in the decrease of glucose consumption and lactate production, but also causes the activation of apoptotic promoting protein Bim and the down-regulation of the anti-apoptotic protein Bcl-xL (Figure 3A). Our results first demonstrate that IFN-α treatment promotes cytochrome c release from mitochondria, indicating that the intrinsic apoptotic pathway can be activated by IFN-α. Yang et al. first showed that IFN-α was able to stimulate the cleavage of caspase 4 in HeLa cells [20]. Our results confirm their observation, and further show that IFN-α-mediated caspase 4 cleavage contributes to the IFN-α-induced apoptosis in HeLa cells. However, IFN-α treatment fails to activate caspase 8 and 10—the important initiators for extrinsic apoptosis—in HeLa cells, indicating that the IFN-α-induced apoptosis may be independent of the extrinsic apoptotic pathway. Our data demonstrate that IFN-α-mediated HeLa cell apoptosis may involve both of the intrinsic and caspase 4-related ER stress-induced apoptotic pathways, however, how these two pathways regulate IFN-α-induced apoptosis needs to be further explored.

## 4. Materials and Methods

### 4.1. Cell Lines, Cell Culture, Chemicals, and Reagents

Human cervical cancercell line (HeLa) was derived from the Cell Culture Center of Institute of Basic Medical Sciences, Chinese Academy of Medical Sciences (Beijing, China). HeLa cells were grown in Dulbecco’s modified Eagle Medium (DMEM) high glucose (Thermo-Fisher Scientific, Waltham, MA, USA) plus 10% fetal bovine serum (FBS) at 37 °C supplemented with 5% CO_2_. Rabbit anti-caspase 3, rabbit anti-Bim, rabbit anti-Bcl-xL, rabbit anti-cytochrome c, mouse anti-β-actin, and rabbit anti-GAPDH were purchased from Santa Cruz Biotechnology (Santa Cruz, CA, USA). Rabbit anti-PARP1 and rabbit anti-cleaved-PARP1 antibodies were purchased from Epitomics Inc. (Burlingame, CA, USA). Mouse anti-caspase 8 and rabbit anti-caspase 10 were purchased from Bioworld Technology Inc. (St. Louis Park, MN, USA). Rabbit anti-caspase 4, rabbit anti-cleaved-caspase 8, rabbit anti-cleaved-caspase 3, and rabbit anti-mtHSP70 were purchased from Proteintech Group Inc. (Rosemont, IL, USA). The peroxidase-conjugated secondary antibodies were purchased from Zhongshan Biotechnology (Beijing, China). Recombinant human interferon α 1 was from ProSpec-Tany TechnoGene Ltd. (Rehovot, Israel). Human tumor necrosis factor-α (TNF-α) and cycloheximide (CHX) were from Sigma-Aldrich Corp (St. Louis, MO, USA). The primers for caspase-4 siRNA, 5′-AGCTTGCCTCAGTCTGAAGGACAACTCGAGTTGTCCTTCAGACTGAGGCG-3′(sense) and 5′-GATCCGCCTCAGTCTGAAGGACAACTCGAGTTGTCCTTCAGACTGAGGCA-3′ (antisense) were synthesized by Sangon Biotech Co., Ltd. (Shanghai, China).

### 4.2. Glucose/Lactate Assay

The levels of glucose and lactate in cell culture medium were detected by Biosen C-Line (EKF Diagnostics, Cardiff, UK). Equal amounts of cells were seeded into a 12-well culture plate. After 24 or 48 h treatment with IFN-α, a 10 μL culture medium from each well was added into the reaction mixture. Detection was performed using Biosen C-Line instrument.

### 4.3. Cell Proliferation Assay

Cell proliferation potential was evaluated by both MTT and CCK-8 assays. Briefly, equal amounts of cells were seeded into a 96-well plate and treated with IFN-α for 24 or 48 h before testing. MTT solution or Cell-Counting Kit-8 (Dojindo Molecular Technologies, Ltd., Kumamoto, Japan) solution was added into each well. After 2.5–3 h incubation, the absorbance at 490 or 450 nm was measured using a microplate reader.

### 4.4. Flow Cytometric Analysis

Cell apoptosis was detected by Annexin V-FITC Apoptosis Detection Kit (Biotool, Beijing, China). After 48 h treatment with IFN-α, the cells were harvested and washed with precooled 1× PBS (phosphate-buffered saline). The cell pellet was resuspended with 50 μL 1× binding buffer followed by adding 5 μL Annexin V-FITC and 5 μL propidiumiodide (PI). The reaction mixture was incubated away from light for 15 min at room temperature. Then, another 150 μL binding buffer was added into the mixture. The reaction product was subjected to flow cytometric analysis by Accuri C6 (BD Biosciences, San Jose, CA, USA).

### 4.5. Mitochondria Isolation

Mitochondria were isolated using Mitochondria Isolation Kit for Cultured Cells (Thermo Scientific Inc., Waltham, MA, USA) as described in the manual.

### 4.6. Real-Time Quantatitive Reverse Transcription PCR (qRT-PCR)

After 48 h incubation, the IFN-α-treated HeLa cells were collected. The cell pellet was lysed with TRIzol (Invitrogen, Carlsbad, CA, USA). Total RNAs were precipitated from the aqueous phase upon chloroform treatment and then subjected to qRT-PCR analysis using One Step SYBR PrimeScript RT-PCR Kit II (Takara Biotechnology, Dalian, China) with primers listed in Table 1. The real-time qRT-PCR was performed using iQ7 system (Bio-Rad Laboratories, Hercules, CA, USA). The reverse transcription was conducted at 42 °C for 5 min. After denaturing at 95 °C for 10 s, the PCR reaction was performed at 95 °C for 5 s and 60 °C for 30 s and repeated for 40 cycles. The dissociation of the reaction products was from 55to 95 °C as the temperature was increased by 0.2 °C per 10 s. The *β-actin* gene expression served as an internal control for normalization.

### 4.7. Western Blot Analysis

The IFN-α-treated HeLa cells and control cells were collected after 48 h incubation. The cell pellets were lysed with lysis buffer containing 1% NP-40, 50 mM Tris-HCl (pH 7.5), 120 mM NaCl, plus proteinase inhibitors. The resolved protein samples by SDS-PAGE were blotted onto Hybond nitrocellular membrane (Amersham Biosciences, Freiburg, Germany). The reaction product was first probed with a primary antibody. After extensively washing, a second antibody conjugated to horseradish peroxidase and specific for the Fc of the first antibody was employed. The reaction products were developed using the chemiluminescence kit (Santa Cruz Biotechnology, Santa Cruz, CA, USA).

### 4.8. Statistical Analysis

Statistical differences were carried out using standard Student’s *t* test (two-tailed, unpaired). The statistical difference was considered to be significant as *p* < 0.05 (*) or *p* < 0.01 (**).

## 5. Conclusions

In the current study, HeLa cells were used as a testing model for the treatment of IFN-α on cervical cancer. We found that IFN-α could markedly inhibit cell proliferation and induce cell apoptosis in HeLa cells. IFN-α activates both the intrinsic mitochondrial pathway and ER stress-induced pathway in HeLa cells. Our results highlight a previously unrecognized role of IFN-α on HeLa cells and may provide a new train of thought for future mechanistic studies.

## Figures and Tables

**Table 1:** Primers used in qRT-PCR analysis.

Gene Name	GenBank ID	Forward Primer (5′→3′)	Reverse Primer (5′→3′)	Size of Product (bp)
β-Actin	BC009275	TCCATCATGAAGTGTGACGT	CTCAGGAGGAGCAATGATCT	161
Caspase 4	EF636667	TTGCTTTCTGCTCTTCAACG	GTGTGATGAAGATAGAGCCCATT	72
